# Supplementary figures and images for: Phytoplasma SAP11 effector destabilization of TCP transcription factors differentially impact development and defence of Arabidopsis versus maize
Source: PLoS Pathog. 2019 Sep 26;15(9):e1008035. doi: 10.1371/journal.ppat.1008035 (PMC6802841; doi:10.1371/journal.ppat.1008035)

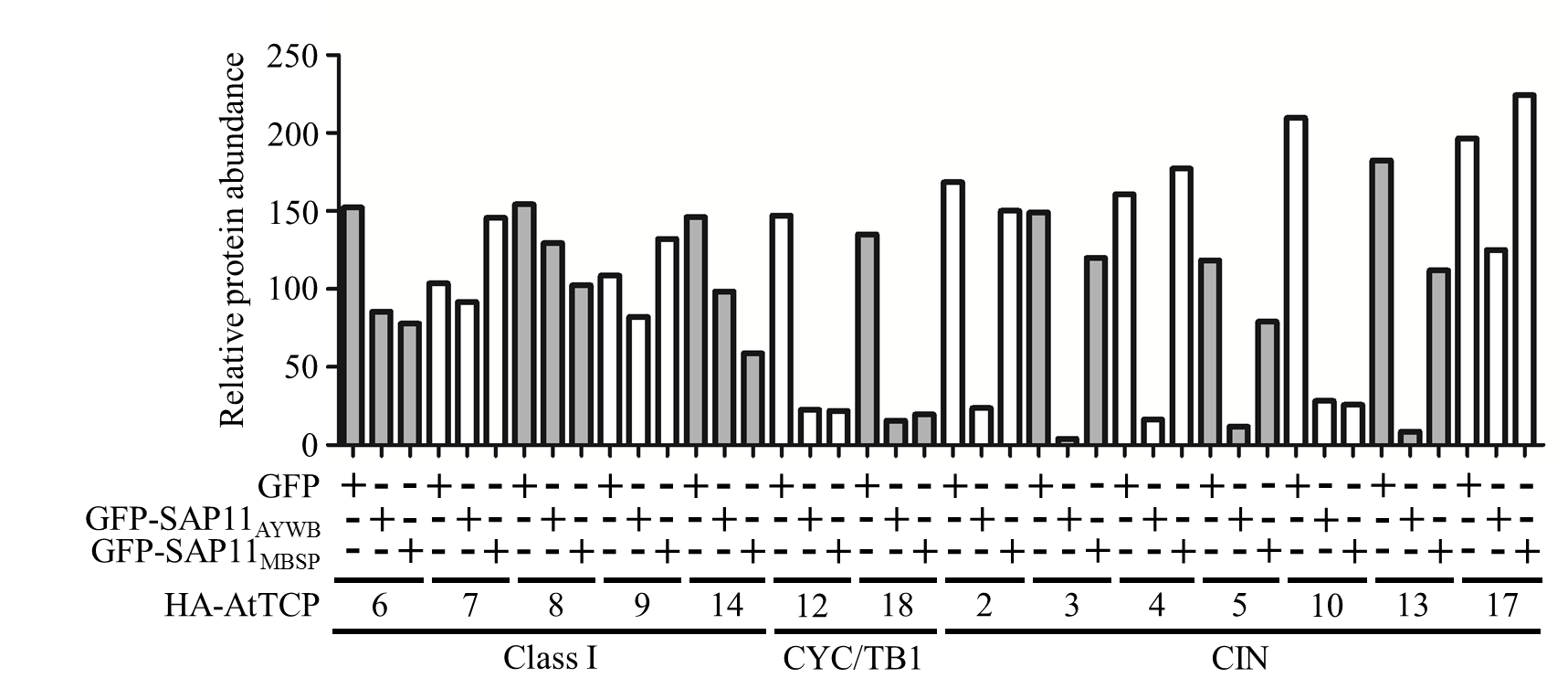

Supplement: S1 Fig — Western blot bands representing HA-TCPs in Fig 1A were quantified with IMAGEJ and presented as relative values corrected by the loading controls. (TIF) [file ppat.1008035.s001.tif]

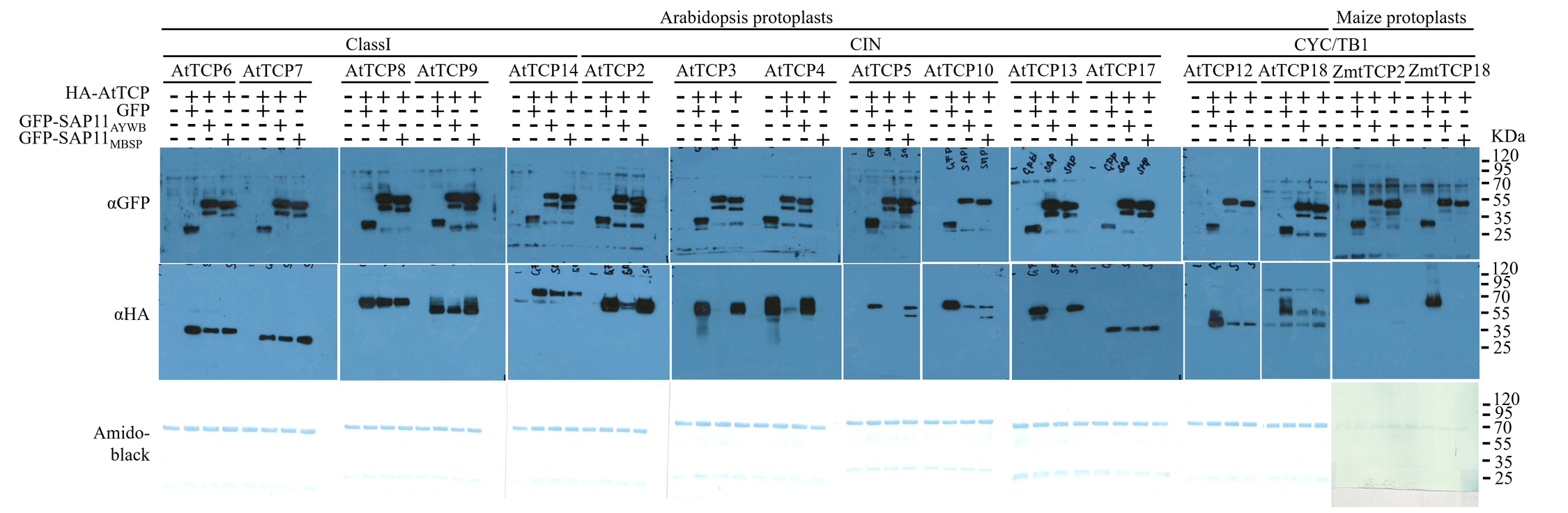

Supplement: S2 Fig — SAP11 or GFP alone and HA-tagged TCPs were detected with specific antibodies to GFP and HA, respectively, as indicated at left of the blots. Loading controls: Amidoblack-stained large RUBISCO subunit. (TIF) [file ppat.1008035.s002.tif]

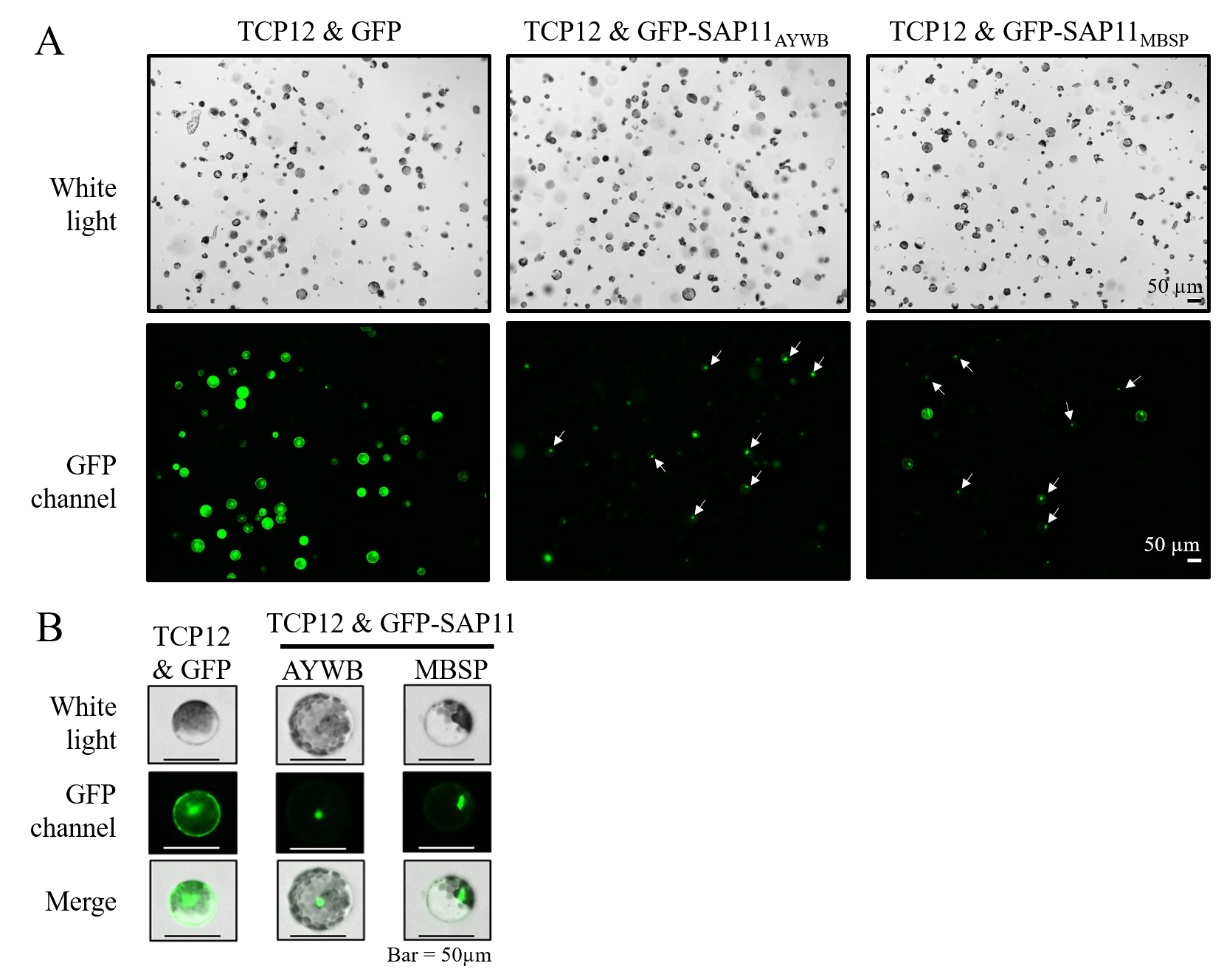

Supplement: S3 Fig — Plasmids for various constructs were introduced into A. thaliana protoplasts and localization of GFP-tagged proteins in the cells observed by confocal microscopy. (A) Images of multiple protoplasts. Green fluorescence indicates the presence of GFP, GFP-SAP11AYWB and GFP-SAP11MBSP as indicated. Arrow heads indicate nuclear localization of the SAP11 proteins. (B) Enlarged images of single protoplast cells of images shown in A. (TIF) [file ppat.1008035.s003.tif]

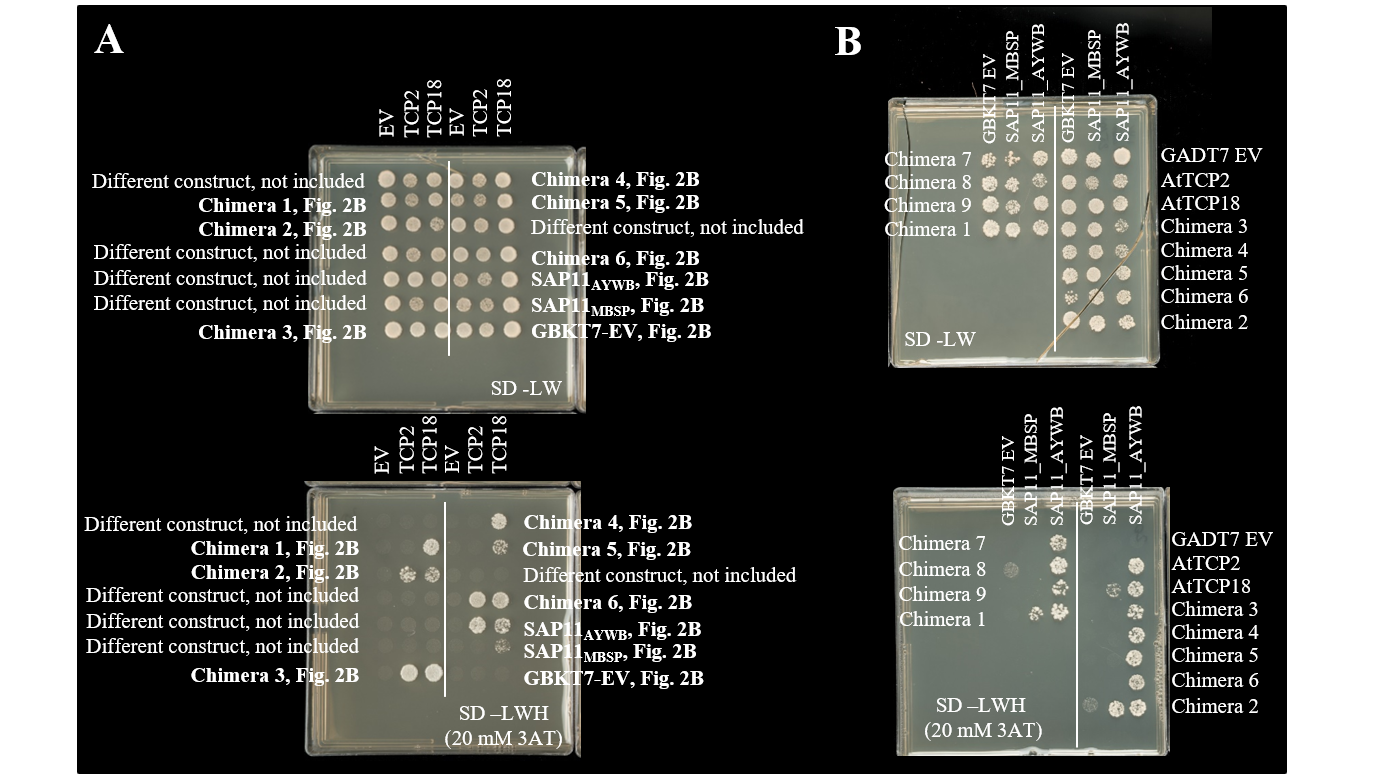

Supplement: S4 Fig — Original plates of yeast two-hybrid experiments used to generate compilations shown in Fig 2B(A) and 2D(B). Abbreviations: EV, Empty vector control; SD-LW, media composition that enables yeast to grow when SAP11 and TCP plasmids are present; SD-LWH (20 mM 3-Amino-1,2,4-triazole (3AT)), selection medium that shows growth of yeast colonies only when SAP11 and TCP interact. (TIF) [file ppat.1008035.s004.tif]

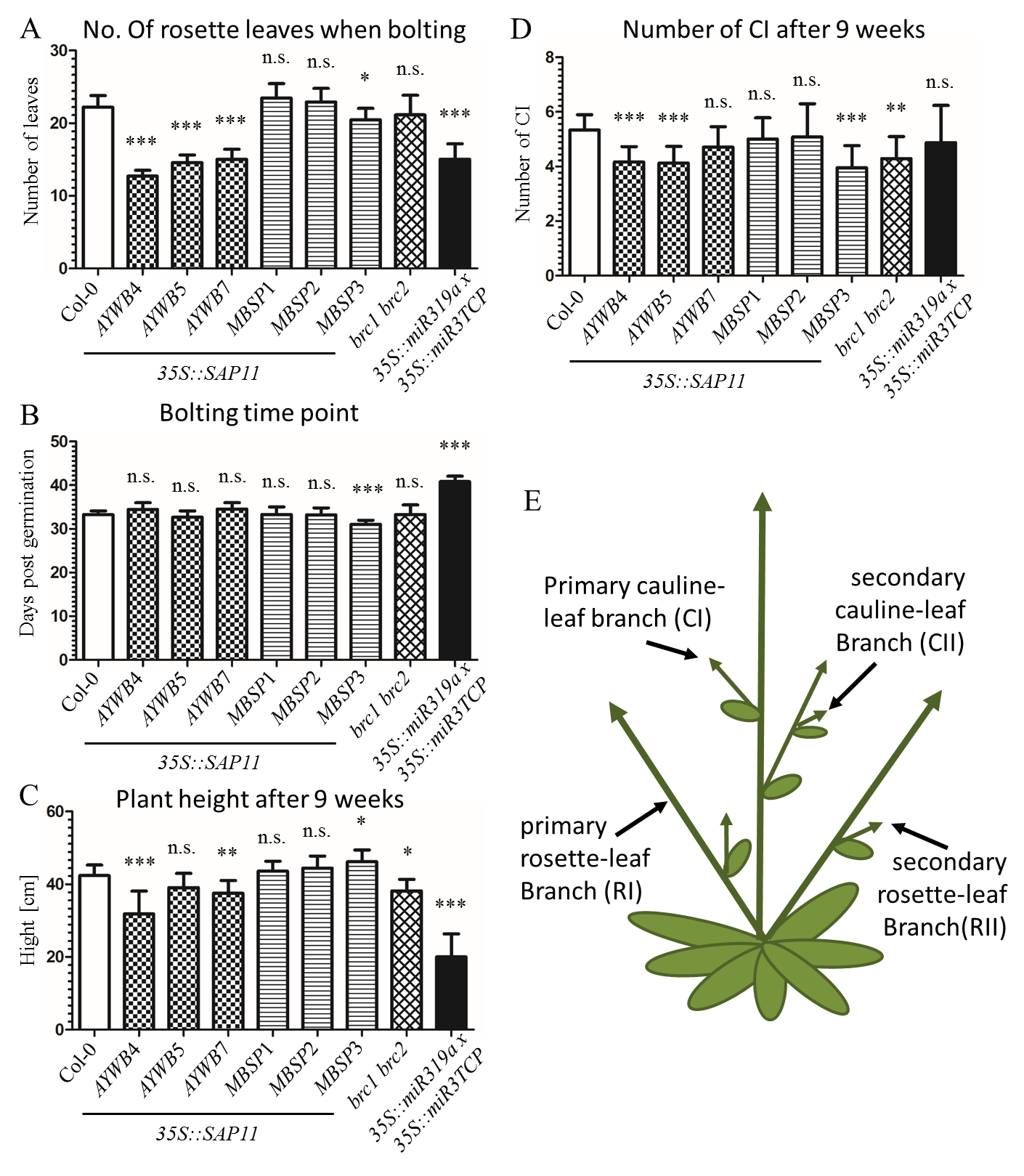

Supplement: S5 Fig — Three independent lines overexpressing either SAP11AYWB or SAP11MBSP were analysed in comparison to Col-0, the brc1 brc2 mutant and 35S::miR319a x 35S::miR3TCP with regard to (A) the number of rosette leaves when first bolting buds appeared at the centre of the leaf rosette, (B) the time point of bolting buds appearance, (C) the plant height and (D) the number of primary cauline-leaf branches (CI). The number of primary rosette-leaf branches (RI) are presented in Fig 1G of the main text. (E) Schematic presentation of Arabidopsis branching. Error bars denote standard errors (n = 24). Asterisks indicate statistically significant differences compared to Col-0. (*, p<0.05, **, p<0.01, ***, p<0.001, student´s t-test); ns, not significant. (TIF) [file ppat.1008035.s005.tif]

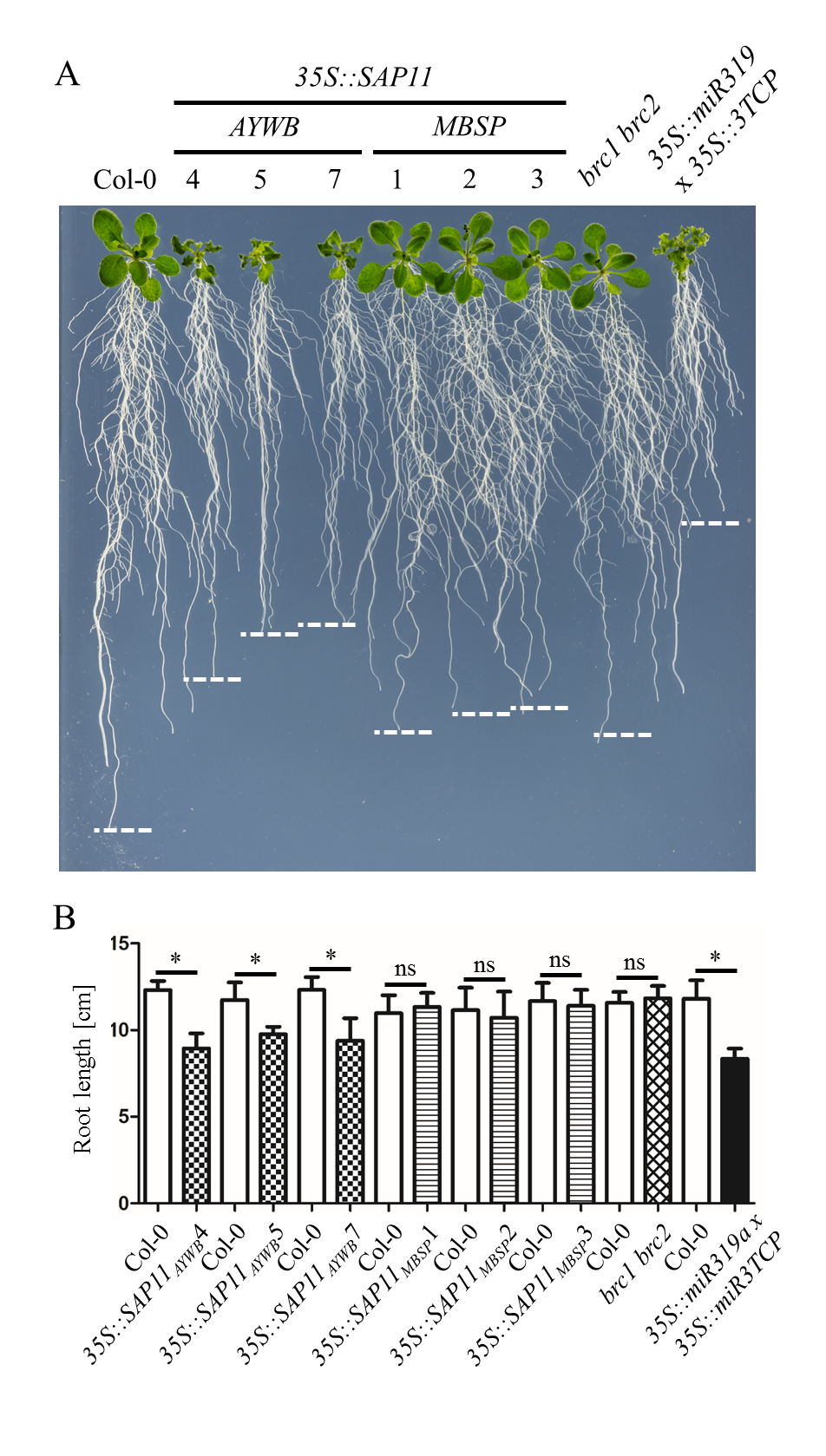

Supplement: S6 Fig — (A) Roots of representative 13 days old 35S::SAP11AYWB and 35S::SAP11MBSP mutants compared to Col-0, the brc1 brc2 mutant and 35S::miR319a x 35S::miR3TCP lines. (B) Root length measurements of indicated mutants compared to Col-0. Error bars denote standard errors (n = 20). Asterisks indicates statistically significant difference (*, p<0.001, student´s t-test); ns, not significant. (TIF) [file ppat.1008035.s006.tif]

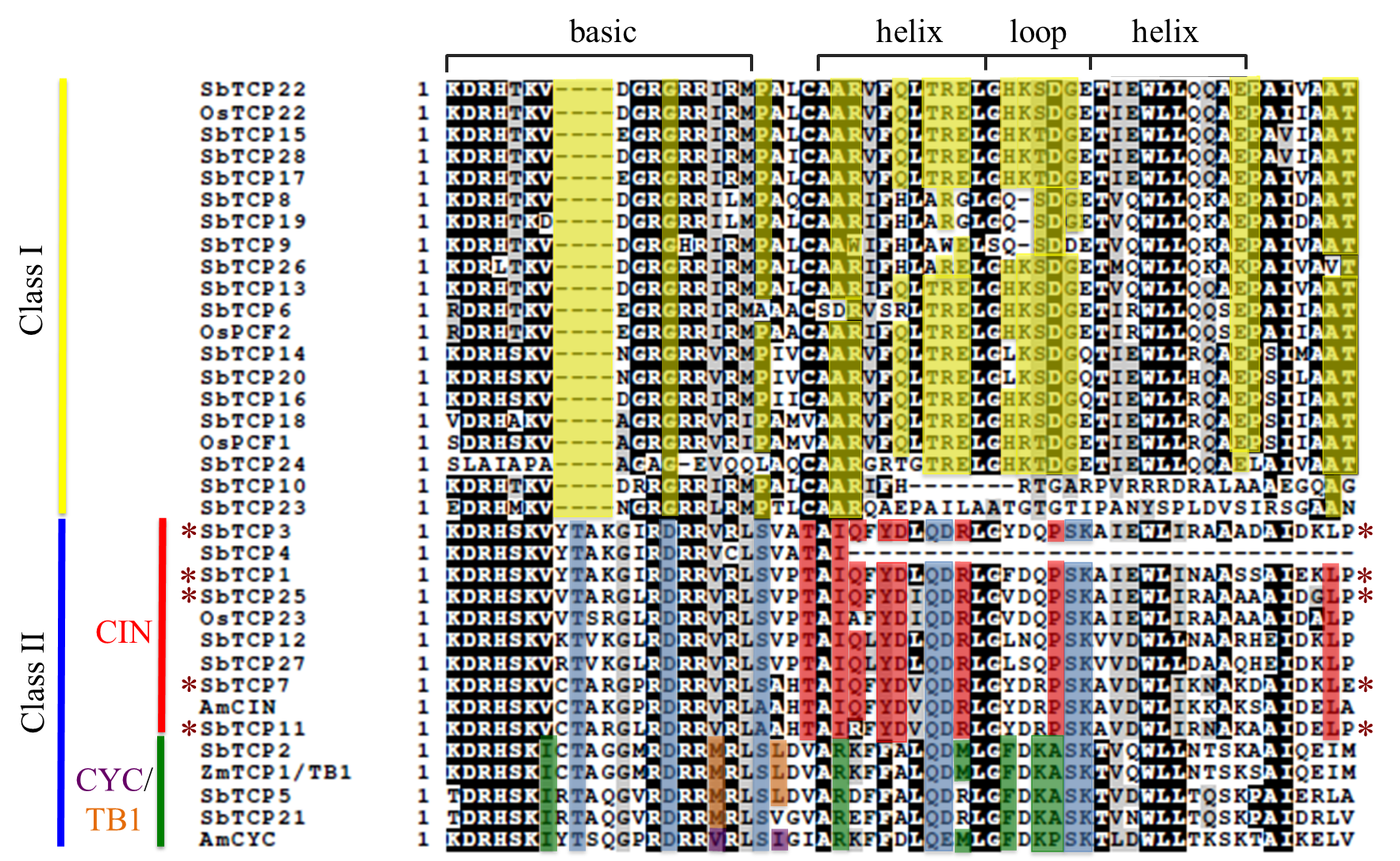

Supplement: S7 Fig — The TCP motifs of 27 SbTCPs (http://grassius.org/grasstfdb.html) were aligned and assigned to the TCP (sub)groups as described in Fig 4. Corresponding gene codes are presented in S4 Table. SbTCP4 carries a truncated TCP-motif at its C-terminus and SbTCP10 and SbTCP23 carry incomplete versions of the TCP-motif within their amino acid sequence. Sequences were aligned using ClustalW (http://www.genome.jp/tools/clustalw/) and visualized using the Boxshade software (http://www.ch.embnet.org/software/BOX_form.html). Asterisks indicate TCPs with potential miR319a target sites identified in their coding gene sequences (S9 Fig). (TIF) [file ppat.1008035.s007.tif]

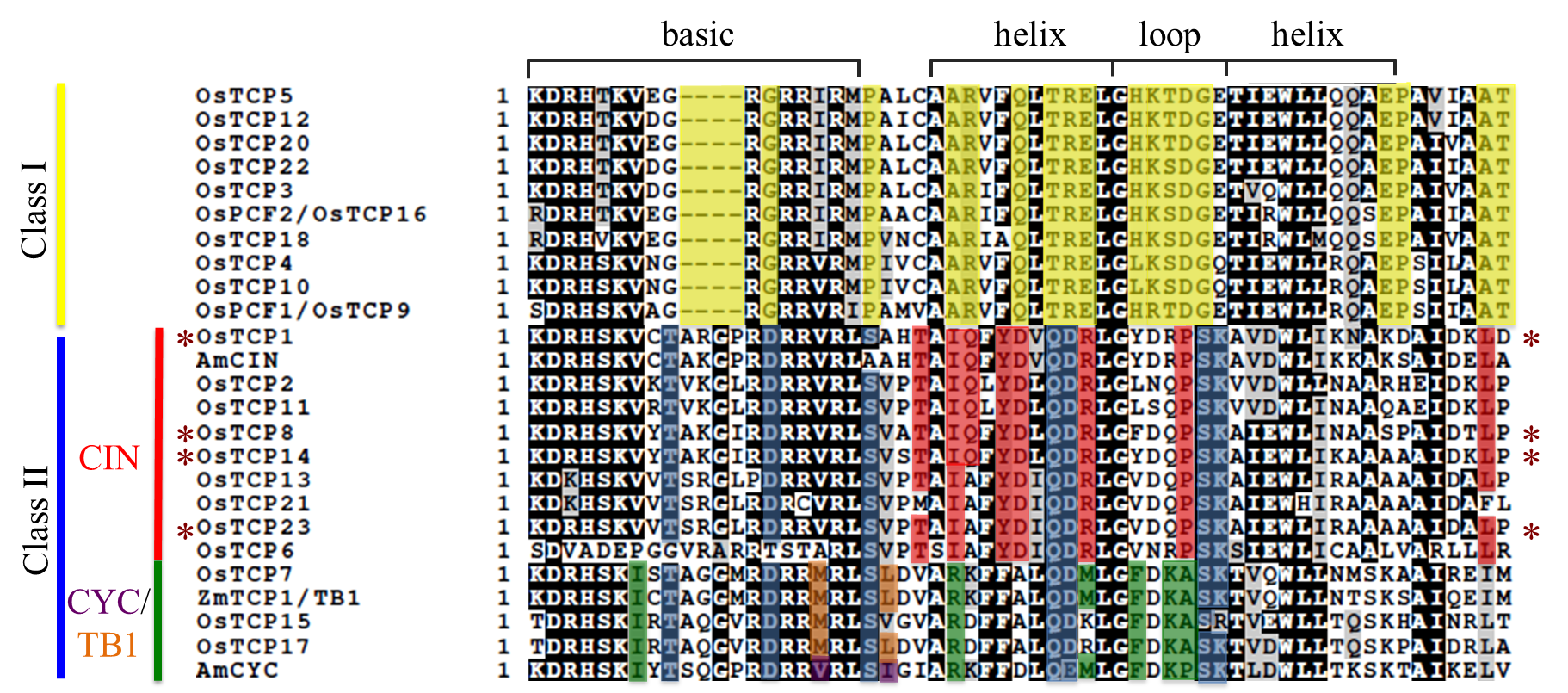

Supplement: S8 Fig — The TCP motifs of 27 OzTCPs (http://grassius.org/grasstfdb.html) were aligned and assigned to the TCP (sub)groups as described in Fig 4. Corresponding gene codes are presented in S4 Table. Sequences were aligned using ClustalW (http://www.genome.jp/tools/clustalw/) and visualized using the Boxshade software (http://www.ch.embnet.org/software/BOX_form.html). Asterisks indicate TCPs with potential miR319a target sites identified in their coding gene sequences (S9 Fig). (TIF) [file ppat.1008035.s008.tif]

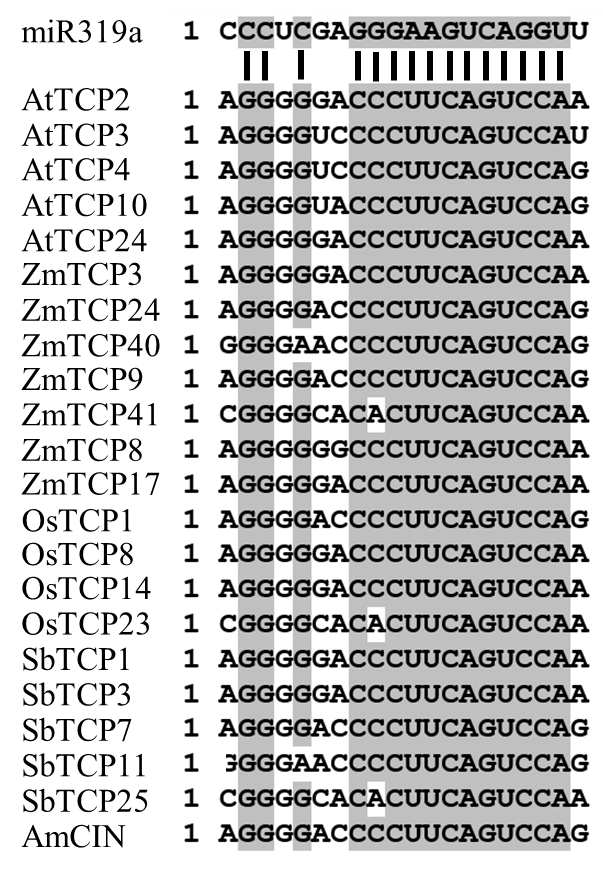

Supplement: S9 Fig — The CDS of the TCPs from Zea mays (Zm), Oryza sativa (Os), Sorghum bicolor (Sb), and of the Antirrhinum majus (Am) CIN-TCP were screened for potential miR319a target sites. They are depicted together with the miR319a binding sites of Arabidopsis thaliana (At) CIN-TCPs [59]. Nucleotides known to be involved in miR319a binding to AtCIN-TCPs are indicated in grey [59]. (TIF) [file ppat.1008035.s009.tif]

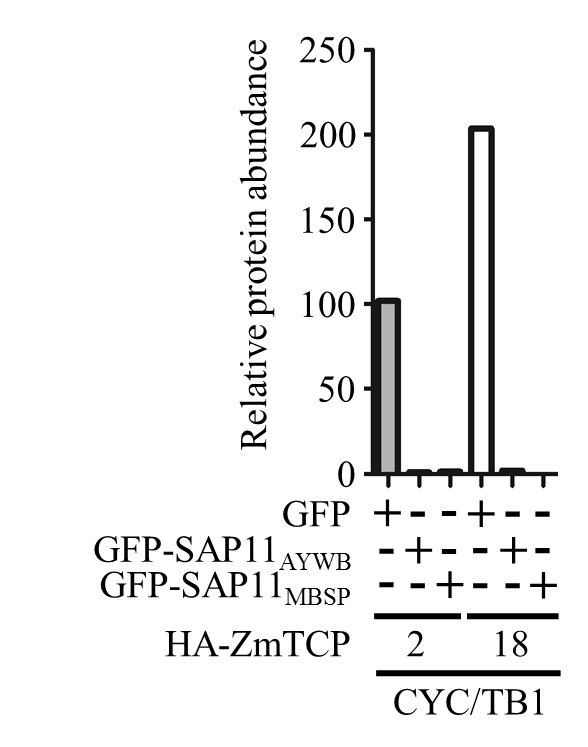

Supplement: S10 Fig — Western blot bands representing HA-TCPs in Fig 5B were quantified with IMAGEJ and presented as relative values corrected by the loading controls. (TIF) [file ppat.1008035.s010.tif]

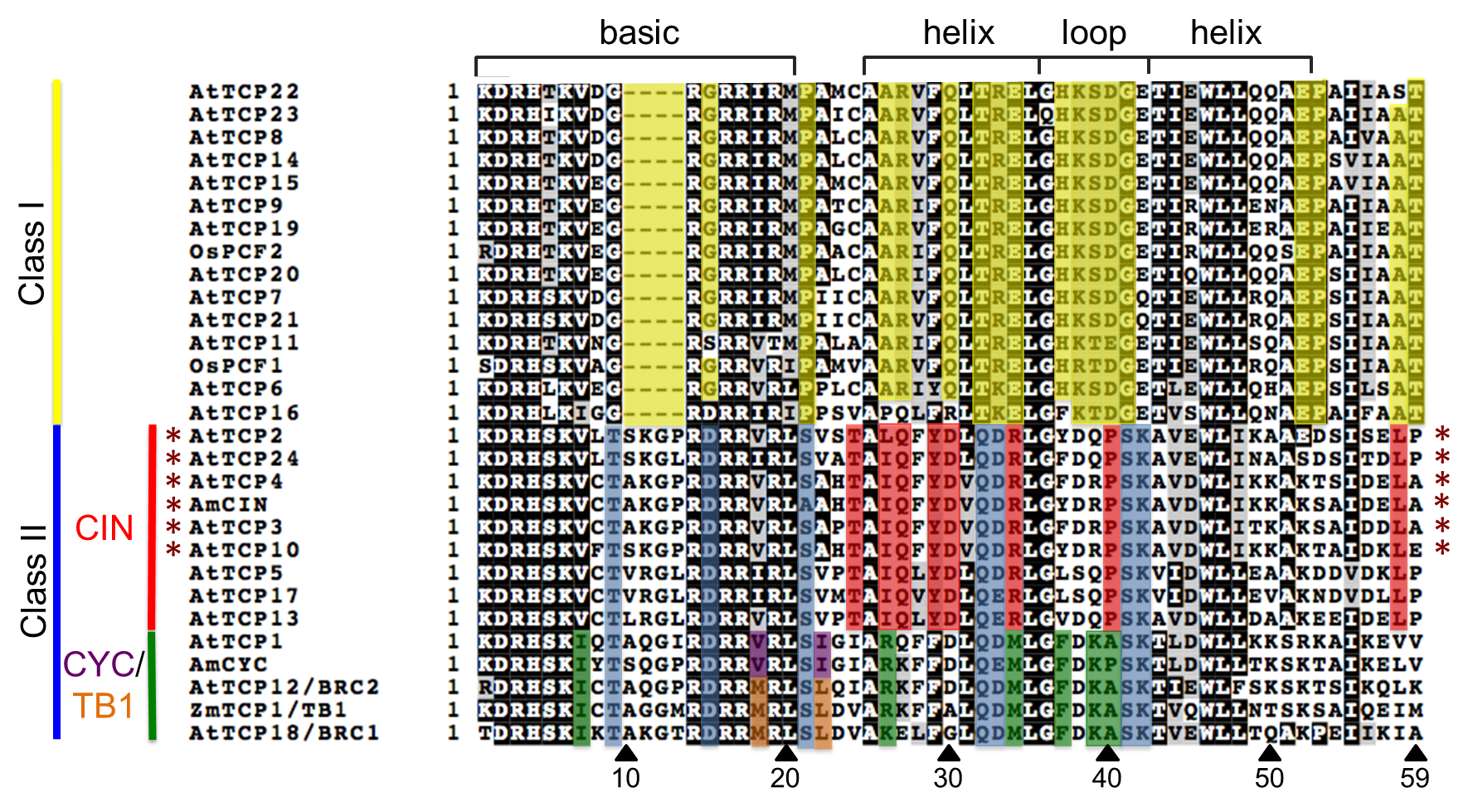

Supplement: S11 Fig — The TCP domains are conserved among TCPs and show consistent differences in amino acid conservation among the TCP (sub)classes, See also legend of Fig 4. Corresponding gene codes are presented in S4 Table. Sequences were aligned using ClustalW (http://www.genome.jp/tools/clustalw/) and visualized using the Boxshade software (http://www.ch.embnet.org/software/BOX_form.html). Asterisks indicate TCPs with miR319a target sites identified in their coding gene sequences (S5 Fig) [59]. (TIF) [file ppat.1008035.s011.tif]

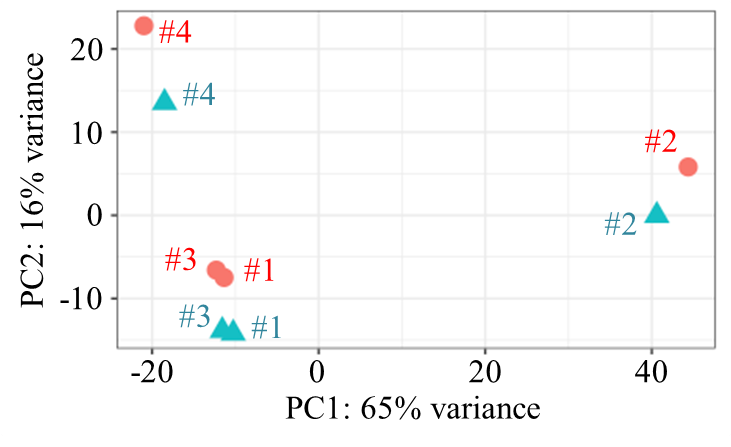

Supplement: S12 Fig — PCA was conducted with normalized read counts of RNA-seq data obtained from M. quadrilineatus-exposed leaves of three A. thaliana Col-0 plants (samples #1, 2 and 3) and 35S::miR319a x 35S::miR3TCP sample #4 generated at the Earlham Institute, Norwich, UK (red circles) and Academia Sinica, Taipei, Taiwan (green triangles). (TIF) [file ppat.1008035.s012.tif]

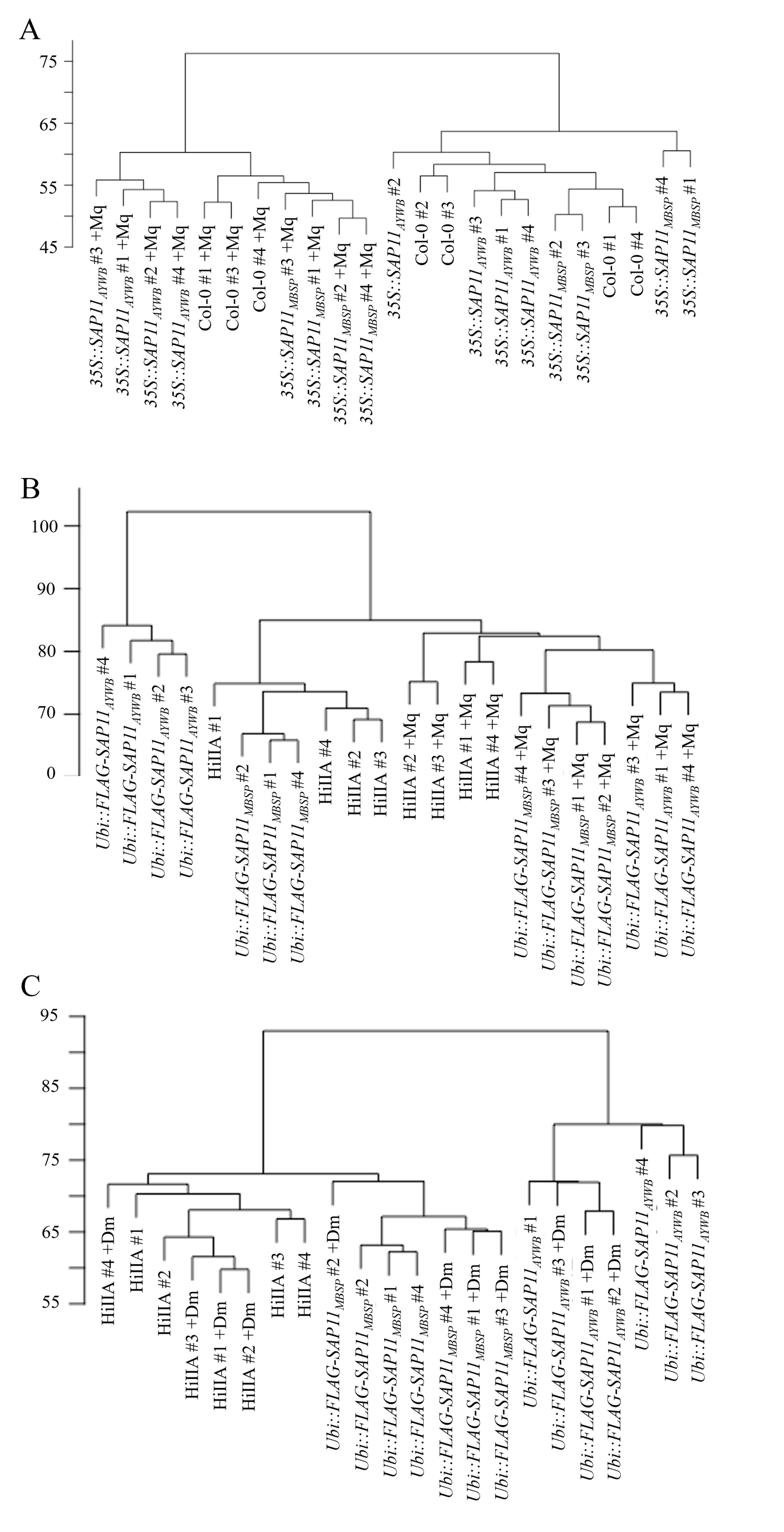

Supplement: S13 Fig — Cluster analysis performed on the matrix of normalized read counts of RNA-seq values from (A) Arabidopsis Col-0, 35S::SAP11AYWB and 35S::SAP11MBSP non-exposed and exposed to M quadrilineatus (+Mq). (B) Z. mays HiIIA, Ubi::FLAG-SAP11AYWB and Ubi::FLAG-SAP11MBSP non-exposed and exposed to M. quadrilineatus (+Mq) and (C) non-exposed and exposed to D. maidis (+Dm). Experiments were done with 35S::SAP11AYWB line 7 [8], 35S::SAP11MBSP line 1, Ubi::FLAG-SAP11AYWB line 1 and Ubi::FLAG-SAP11MBSP line 1. (TIF) [file ppat.1008035.s013.tif]
