# Supplementary material for: Phytoplasma SAP11 effector destabilization of TCP transcription factors differentially impact development and defence of Arabidopsis versus maize
Source: PLoS Pathog. 2019 Sep 26;15(9):e1008035. doi: 10.1371/journal.ppat.1008035 (PMC6802841; doi:10.1371/journal.ppat.1008035)
Supplement: S7 Table — (DOCX) [file ppat.1008035.s020.docx]

**S7 Table.** **Oligonucleotide sequences (5´ > 3´) for cloning**.

| **Oligonucleotide** | **Sequence (5 -> 3)** |
| --- | --- |
| attB1 adapter | GGGGACAAGTTTGTACAAAAAAGCAGGCT |
| attB2 adapter | GGGGACCACTTTGTACAAGAAAGCTGGGT |
| AtTCP6 forward | AAAAAGCAGGCTCCACCATGGTCATGGAGCCCAAGAAGAACC |
| AtTCP6 reverese | AGAAAGCTGGGTGTCATTATGAACCATTTTCCTCTGCACTC |
| AtTCP8 forward | AAAAAGCAGGCTCCACCATGGATCTCTCCGACATCC |
| AtTCP8 reverse | AGAAAGCTGGGTGTCAGAGCTATTTGAGTTCTC |
| AtTCP9 forward | AAAAAGCAGGCTCCACCATGGCAATTCAGAAGC |
| AtTCP9 reverse | AGAAAGCTGGGTGTCAGTGGTTCGATGACCGTG |
| AtTCP12 forward | AAAAAGCAGGCTCCACCATGTTTCCTTCTCTAGATACCAATGG |
| AtTCP12 reverse | AGAAAGCTGGGTGTCATCAGTAGCAGAGATAATCATATAG |
| AtTCP14 forward | AAAAAGCAGGCTCCACCATGCAAAAGCCAACATCAAG |
| AtTCP14 reverse | AGAAAGCTGGGTGCTAATCTTGCTGATCCTCCTC |
| AtTCP18 forward | AAAAAGCAGGCTCCACCATGAACAACAACATTTTCAGTACTACTACCACC |
| AtTCP18 reverse | AGAAAGCTGGGTGTTATCAATACATGTTTTGATAGTTGTGCATGAGGTC |
| Stop-GFP forward | GGGGACAAGTTTGTACAAAAAAGCAGGCTGATGAATGGTGAGCAAGGGCG |
| Stop-GFP reverse | CACTTTGTACAAGAAAGCTGGGTGTCACTTGTACAGCTCGTCCATGC |
