# Supplementary material for: Phytoplasma SAP11 effector destabilization of TCP transcription factors differentially impact development and defence of Arabidopsis versus maize
Source: PLoS Pathog. 2019 Sep 26;15(9):e1008035. doi: 10.1371/journal.ppat.1008035 (PMC6802841; doi:10.1371/journal.ppat.1008035)
Supplement: S8 Table — Nucleotide sequences for gene syntheses of SAP11MBSP and SAP11-chimeras for expression in Arabidopsis thaliana and yeast and of the TCP domains from ZmTCP33, AtTCP2, AtTCP18 and chimeras of AtTCP2 and AtTCP18 TCP domains for expression in yeast. (DOCX) [file ppat.1008035.s021.docx]

**S8 Table.** **Synthesized CDS (underlined) flanked by gateway compatible attL1 and attL2 sites.** Nucleotide sequences for gene syntheses of *SAP11_MBSP_* and SAP11-chimeras for expression in *Arabidopsis thalina* and yeast and of the TCP domains from *ZmTCP33*, *AtTCP2*, *AtTCP18* and chimeras of *AtTCP2* and *AtTCP18* TCP domains for expression in yeast.

| **Codon optimized *SAP11_MBSP_* without NLS for expression in *A.thaliana* and yeast** |
| --- |
| GCCCCAAATAATGATTTTATTTTGACTGATAGTGACCTGTTCGTTGCAACAAATTGATGAGCAATGCTTTTTTATAATGCCAACTTTGTACAAAAAAGCAGGCTCCGGTACCGCCATGTCTCCTAAGAAAGAAGATAGAGGAAAGAATGTTGCTACATCAAAGGAAAAAGAGACTCTTACAAAGGAAGAGGTGAAAAGGTTTTTCGAGTACCATAAGACCTTCGAAACTTACAGTGATGAGGATAAGATAAAAATTATCGAAAAGATCACCGATCCTGAGGTTTCAAAACTTTTGGATGAATACAATGAGAAGAAAAGAAAGTCTTCAAAAGAAGAGAGTTCTTCTAGTAAGAAACCAGATAACTCTAAGAAATAAGGCAAGCTTGACCCAGCTTTCTTGTACAAAGTTGGCATTATAAAAAATAATTGCTCATCAATTTGTTGCAACGAACAGGTCACTATCAGTCAAAATAAAATCATTATTTGCCATC |
| **SAP11-Chimera 1: chimeric SAP11 without NLS of *SAP11_AYWB_* and *SAP11_MBSP_* used in Y2H analysis** |
| GCCCCAAATAATGATTTTATTTTGACTGATAGTGACCTGTTCGTTGCAACAAATTGATGAGCAATGCTTTTTTATAATGCCAACTTTGTACAAAAAAGCAGGCTCCGGTACCGCCTCACCTAAAAAAGAAGATCGCGGAAAAAATGTTGCAACTTCAAAAGAAAAAGAAACACTAACTAAAGAAGAAGTGAAACGTTTTTTTGAATACCATAAAACATTTGAAACATATTCTGATGAAGACAAAATTAAAATTATTGAAAAAATTACCGACCCAGAAGTAATGGAAATATTAAAACAAAAAGCCGAAGAGGAAACGAAAAATTTAAAGGAAGAAAGTTCTTCTAGCAAAAAACCTGATAATTCAAAAAAATAAGGCAAGCTTGACCCAGCTTTCTTGTACAAAGTTGGCATTATAAAAAATAATTGCTCATCAATTTGTTGCAACGAACAGGTCACTATCAGTCAAAATAAAATCATTATTTGCCATC |
| **SAP11-Chimera 2: chimeric SAP11 without NLS of *SAP11_AYWB_* and *SAP11_MBSP_* used in Y2H analysis** |
| GCCCCAAATAATGATTTTATTTTGACTGATAGTGACCTGTTCGTTGCAACAAATTGATGAGCAATGCTTTTTTATAATGCCAACTTTGTACAAAAAAGCAGGCTCCGGTACCGCCTCACCTAAAAAAGAATCTAGTGATAAAAATGTTGCAACTTCAAAAGAAAAAGAAACACTAACTAAAGAAGATATAAAAAGATTTTATACAATACATAAAGAATTTAAAGAATATTCAATTGAAAAAAATAATGAAATTATAAAAATTTTAGAAAACCCTGAATTAATGGAAATATTAAAACAAAAAGCCGAAGAGGAAACGAAAAATTTAAAAGAAGAAGGTTCTTCTTCAAAACAACCTGATGATTCTAAAAAATAAGGCAAGCTTGACCCAGCTTTCTTGTACAAAGTTGGCATTATAAAAAATAATTGCTCATCAATTTGTTGCAACGAACAGGTCACTATCAGTCAAAATAAAATCATTATTTGCCATC |
| **SAP11-Chimera 3: chimeric SAP11 without NLS of *SAP11_AYWB_* and *SAP11_MBSP_* used in Y2H analysis** |
| GCCCCAAATAATGATTTTATTTTGACTGATAGTGACCTGTTCGTTGCAACAAATTGATGAGCAATGCTTTTTTATAATGCCAACTTTGTACAAAAAAGCAGGCTCCGGTACCGCCTCACCTAAAAAAGAATCTAGTGATAAAAATGTTGCAACTTCAAAAGAAAAAGAAACACTAACTAAAGAAGATATAAAAAGATTTTATACAATACATAAAGAATTTAAAGAATATTCAATTGAAAAAAATAATGAAATTATAAAAATTTTAGAAAACCCTGAATTAATGGAAATATTAAAACAAAAAGCCGAAGAGGAAACGAAAAATTTAAAGGAAGAAAGTTCTTCTAGCAAAAAACCTGATAATTCAAAAAAATAAGGCAAGCTTGACCCAGCTTTCTTGTACAAAGTTGGCATTATAAAAAATAATTGCTCATCAATTTGTTGCAACGAACAGGTCACTATCAGTCAAAATAAAATCATTATTTGCCATC |
| **SAP11-Chimera 4: chimeric SAP11 without NLS of *SAP11_AYWB_* and *SAP11_MBSP_* used in Y2H analysis** |
| GCCCCAAATAATGATTTTATTTTGACTGATAGTGACCTGTTCGTTGCAACAAATTGATGAGCAATGCTTTTTTATAATGCCAACTTTGTACAAAAAAGCAGGCTCCGGTACCGCCTCACCTAAAAAAGAATCTAGTGATAAAAATGTTGCAACTTCAAAAGAAAAAGAAACACTAACTAAAGAAGAAGTGAAACGTTTTTTTGAATACCATAAAACATTTGAAACATATTCTGATGAAGACAAAATTAAAATTATTGAAAAAATTACCGACCCAGAAGTAATGGAAATATTAAAACAAAAAGCCGAAGAGGAAACGAAAAATTTAAAAGAAGAAGGTTCTTCTTCAAAACAACCTGATGATTCTAAAAAATAAGGCAAGCTTGACCCAGCTTTCTTGTACAAAGTTGGCATTATAAAAAATAATTGCTCATCAATTTGTTGCAACGAACAGGTCACTATCAGTCAAAATAAAATCATTATTTGCCATC |
| **SAP11-Chimera 5: chimeric SAP11 without NLS of *SAP11_AYWB_* and *SAP11_MBSP_* used in Y2H analysis** |
| GCCCCAAATAATGATTTTATTTTGACTGATAGTGACCTGTTCGTTGCAACAAATTGATGAGCAATGCTTTTTTATAATGCCAACTTTGTACAAAAAAGCAGGCTCCGGTACCGCCTCACCTAAAAAAGAATCTAGTGATAAAAAAAGAGATATTCCGAAAATTAATAAATCAGAAGAAAAAAACAAAAAACAAAAAGAAGAAGTGAAACGTTTTTTTGAATACCATAAAACATTTGAAACATATTCTGATGAAGACAAAATTAAAATTATTGAAAAAATTACCGACCCAGAAGTAATGGAAATATTAAAACAAAAAGCCGAAGAGGAAACGAAAAATTTAAAAGAAGAAGGTTCTTCTTCAAAACAACCTGATGATTCTAAAAAATAAGGCAAGCTTGACCCAGCTTTCTTGTACAAAGTTGGCATTATAAAAAATAATTGCTCATCAATTTGTTGCAACGAACAGGTCACTATCAGTCAAAATAAAATCATTATTTGCCATC |
| **SAP11-Chimera 6: chimeric SAP11 without NLS of *SAP11_AYWB_* and *SAP11_MBSP_* used in Y2H analysis** |
| GCCCCAAATAATGATTTTATTTTGACTGATAGTGACCTGTTCGTTGCAACAAATTGATGAGCAATGCTTTTTTATAATGCCAACTTTGTACAAAAAAGCAGGCTCCGGTACCGCCTCACCTAAAAAAGAAGATCGCGGAAAAAATGTTGCAACTTCAAAAGAAAAAGAAACACTAACTAAAGAAGATATAAAAAGATTTTATACAATACATAAAGAATTTAAAGAATATTCAATTGAAAAAAATAATGAAATTATAAAAATTTTAGAAAACCCTGAATTAATGGAAATATTAAAACAAAAAGCCGAAGAGGAAACGAAAAATTTAAAGGAAGAAAGTTCTTCTAGCAAAAAACCTGATAATTCAAAAAAATAAGGCAAGCTTGACCCAGCTTTCTTGTACAAAGTTGGCATTATAAAAAATAATTGCTCATCAATTTGTTGCAACGAACAGGTCACTATCAGTCAAAATAAAATCATTATTTGCCATC |
| **Sequence of TCP domain *AtTCP2* used in Y2H analysis** |
| GCCCCAAATAATGATTTTATTTTGACTGATAGTGACCTGTTCGTTGCAACAAATTGATGAGCAATGCTTTTTTATAATGCCAACTTTGTACAAAAAAGCAGGCTCCGGTACCGCCAAAGATAGACATTCTAAAGTTTTGACTTCCAAGGGTCCAAGAGATAGAAGAGTTAGATTGTCTGTTTCTACCGCCTTGCAATTTTACGACTTGCAAGATAGATTGGGTTACGACCAACCATCTAAAGCTGTTGAATGGTTGATTAAGGCTGCCGAAGATTCCATTTCTGAATTGCCATAAGGCAAGCTTGACCCAGCTTTCTTGTACAAAGTTGGCATTATAAAAAATAATTGCTCATCAATTTGTTGCAACGAACAGGTCACTATCAGTCAAAATAAAATCATTATTTGCCATC |
| **Sequence of TCP domain of *AtTCP18* used in Y2H analysis** |
| GCCCCAAATAATGATTTTATTTTGACTGATAGTGACCTGTTCGTTGCAACAAATTGATGAGCAATGCTTTTTTATAATGCCAACTTTGTACAAAAAAGCAGGCTCCGGTACCGCCACTGATAGACATTCTAAAATCAAAACTGCCAAGGGTACTAGAGATAGAAGAATGAGATTGTCCTTGGATGTCGCCAAAGAATTATTCGGTTTACAAGACATGTTGGGTTTCGATAAGGCTTCTAAAACTGTCGAATGGTTGTTGACTCAAGCCAAGCCAGAAATTATCAAGATTGCCTGAGGCAAGCTTGACCCAGCTTTCTTGTACAAAGTTGGCATTATAAAAAATAATTGCTCATCAATTTGTTGCAACGAACAGGTCACTATCAGTCAAAATAAAATCATTATTTGCCATC |
| **TCP-Chimera 1: chimeric TCP domain of *AtTCP2* and *AtTCP18* used in Y2H analysis** |
| GCCCCAAATAATGATTTTATTTTGACTGATAGTGACCTGTTCGTTGCAACAAATTGATGAGCAATGCTTTTTTATAATGCCAACTTTGTACAAAAAAGCAGGCTCCGGTACCGCCACTGATAGACATTCTAAAATCAAAACTGCCAAGGGTACTAGAGATAGAAGAATGAGATTGTCCGTTTCTACCGCCAAAGAATTATTCGGTTTACAAGACATGTTGGGTTTCGATAAGGCTTCTAAAACTGTCGAATGGTTGTTGACTCAAGCCAAGGATTCCATTTCTGAATTGCCATAAGGCAAGCTTGACCCAGCTTTCTTGTACAAAGTTGGCATTATAAAAAATAATTGCTCATCAATTTGTTGCAACGAACAGGTCACTATCAGTCAAAATAAAATCATTATTTGCCATC |
| **TCP-Chimera 2: chimeric TCP domain of *AtTCP2* and *AtTCP18* used in Y2H analysis** |
| GCCCCAAATAATGATTTTATTTTGACTGATAGTGACCTGTTCGTTGCAACAAATTGATGAGCAATGCTTTTTTATAATGCCAACTTTGTACAAAAAAGCAGGCTCCGGTACCGCCAAAGATAGACATTCTAAAGTTTTGACTTCCAAGGGTCCAAGAGATAGAAGAGTTAGATTGTCTGTTTCTACCGCCAAAGAATTATTCGGTTTACAAGACATGTTGGGTTTCGATAAGGCTTCTAAAACTGTCGAATGGTTGTTGACTCAAGCCAAGGATTCCATTTCTGAATTGCCATAAGGCAAGCTTGACCCAGCTTTCTTGTACAAAGTTGGCATTATAAAAAATAATTGCTCATCAATTTGTTGCAACGAACAGGTCACTATCAGTCAAAATAAAATCATTATTTGCCAT |
| **TCP-Chimera 3: chimeric TCP domain of *AtTCP2* and *AtTCP18* used in Y2H analysis** |
| GCCCCAAATAATGATTTTATTTTGACTGATAGTGACCTGTTCGTTGCAACAAATTGATGAGCAATGCTTTTTTATAATGCCAACTTTGTACAAAAAAGCAGGCTCCGGTACCGCCACTGATAGACATTCTAAAATCAAAACTGCCAAGGGTACTAGAGATAGAAGAATGAGATTGTCTGTTTCTACCGCCTTGCAATTTTACGACTTGCAAGATAGATTGGGTTACGACCAACCATCTAAAGCTGTTGAATGGTTGATTAAGGCTGCCGAAGATTCCATTTCTGAATTGCCATAAGGCAAGCTTGACCCAGCTTTCTTGTACAAAGTTGGCATTATAAAAAATAATTGCTCATCAATTTGTTGCAACGAACAGGTCACTATCAGTCAAAATAAAATCATTATTTGCCATC |
| **TCP-Chimera 4: chimeric TCP domain of *AtTCP2* and *AtTCP18* used in Y2H analysis** |
| GCCCCAAATAATGATTTTATTTTGACTGATAGTGACCTGTTCGTTGCAACAAATTGATGAGCAATGCTTTTTTATAATGCCAACTTTGTACAAAAAAGCAGGCTCCGGTACCGCCAAAGATAGACATTCTAAAGTTTTGACTTCCAAGGGTCCAAGAGATAGAAGAGTTAGATTGTCTGTTTCCACCGCTAAAGAATTATTCGGTTTACAAGACATGTTGGGTTACGACCAACCATCTAAAGCTGTTGAATGGTTGATTAAGGCTGCCGAAGATTCCATTTCTGAATTGCCATAAGGCAAGCTTGACCCAGCTTTCTTGTACAAAGTTGGCATTATAAAAAATAATTGCTCATCAATTTGTTGCAACGAACAGGTCACTATCAGTCAAAATAAAATCATTATTTGCCATC |
| **TCP-Chimera 5: chimeric TCP domain of *AtTCP2* and *AtTCP18* used in Y2H analysis** |
| GCCCCAAATAATGATTTTATTTTGACTGATAGTGACCTGTTCGTTGCAACAAATTGATGAGCAATGCTTTTTTATAATGCCAACTTTGTACAAAAAAGCAGGCTCCGGTACCGCCAAAGATAGACATTCTAAAGTTTTGACTTCCAAGGGTCCAAGAGATAGAAGAGTTAGATTGTCTGTTTCTACCGCCTTGCAATTTTACGACTTGCAAGATAGATTGGGTTTCGATAAGGCTTCTAAAGCTGTTGAATGGTTGATTAAGGCTGCCGAAGATTCCATTTCTGAATTGCCATAAGGCAAGCTTGACCCAGCTTTCTTGTACAAAGTTGGCATTATAAAAAATAATTGCTCATCAATTTGTTGCAACGAACAGGTCACTATCAGTCAAAATAAAATCATTATTTGCCATC |
| **TCP-Chimera 6: chimeric TCP domain of *AtTCP2* and *AtTCP18* used in Y2H analysis** |
| GCCCCAAATAATGATTTTATTTTGACTGATAGTGACCTGTTCGTTGCAACAAATTGATGAGCAATGCTTTTTTATAATGCCAACTTTGTACAAAAAAGCAGGCTCCGGTACCGCCAAAGATAGACATTCTAAAGTTTTGACTTCCAAGGGTCCAAGAGATAGAAGAGTTAGATTGTCTGTTTCTACCGCCTTGCAATTTTACGACTTGCAAGATAGATTGGGTTACGACCAACCATCTAAAGTTGAATGGTTGTTGACTCAAGCCAAGGACTCTATTTCTGAATTGCCATGAGGCAAGCTTGACCCAGCTTTCTTGTACAAAGTTGGCATTATAAAAAATAATTGCTCATCAATTTGTTGCAACGAACAGGTCACTATCAGTCAAAATAAAATCATTATTTGCCATC |
| **TCP-Chimera 7: chimeric TCP domain of *AtTCP2* and *AtTCP18* used in Y2H analysis** |
| GCCCCAAATAATGATTTTATTTTGACTGATAGTGACCTGTTCGTTGCAACAAATTGATGAGCAATGCTTTTTTATAATGCCAACTTTGTACAAAAAAGCAGGCTCCGGTACCGCCAAAGATAGACATTCTAAAGTTTTGACTTCCAAGGGTCCAAGAGATAGAAGAGTTAGATTGTCTGTTTCTACCGCCTTGCAATTTTACGACTTGCAAGATAGATTGGGTTTCGATAAGGCTTCTAAAACTGTCGAATGGTTGTTGACTCAAGCCAAGGATTCCATTTCTGAATTGCCATAAGGCAAGCTTGACCCAGCTTTCTTGTACAAAGTTGGCATTATAAAAAATAATTGCTCATCAATTTGTTGCAACGAACAGGTCACTATCAGTCAAAATAAAATCATTATTTGCCATC |
| **TCP-Chimera 8: chimeric TCP domain of *AtTCP2* and *AtTCP18* used in Y2H analysis** |
| GCCCCAAATAATGATTTTATTTTGACTGATAGTGACCTGTTCGTTGCAACAAATTGATGAGCAATGCTTTTTTATAATGCCAACTTTGTACAAAAAAGCAGGCTCCGGTACCGCCAAAGATAGACATTCTAAAGTTTTGACTTCCAAGGGTCCAAGAGATAGAAGAGTTAGATTGTCTGTTTCTACCGCCAAAGAATTATTCGGTTTACAAGACATGTTGGGTTTCGATAAGGCTTCTAAAGCTGTTGAATGGTTGATTAAGGCTGCCGAAGATTCCATTTCTGAATTGCCATAAGGCAAGCTTGACCCAGCTTTCTTGTACAAAGTTGGCATTATAAAAAATAATTGCTCATCAATTTGTTGCAACGAACAGGTCACTATCAGTCAAAATAAAATCATTATTTGCCATC |
| **TCP-Chimera 9: chimeric TCP domain of *AtTCP2* and *AtTCP18* used in Y2H analysis** |
| GCCCCAAATAATGATTTTATTTTGACTGATAGTGACCTGTTCGTTGCAACAAATTGATGAGCAATGCTTTTTTATAATGCCAACTTTGTACAAAAAAGCAGGCTCCGGTACCGCCACTGATAGACATTCTAAAATCAAAACTGCCAAGGGTACTAGAGATAGAAGAATGAGATTGTCCGTTTCTACCGCCAAAGAATTATTCGGTTTACAAGACATGTTGGGTTACGACCAACCATCTAAAACTGTCGAATGGTTGTTGACTCAAGCCAAGGATTCCATTTCTGAATTGCCATAAGGCAAGCTTGACCCAGCTTTCTTGTACAAAGTTGGCATTATAAAAAATAATTGCTCATCAATTTGTTGCAACGAACAGGTCACTATCAGTCAAAATAAAATCATTATTTGCCATC |
| **TCP domain of *ZmTCP33* used in Y2H analysis** |
| GCCCCAAATAATGATTTTATTTTGACTGATAGTGACCTGTTCGTTGCAACAAATTGATGAGCAATGCTTTTTTATAATGCCAACTTTGTACAAAAAAGCAGGCTCCGGTACCGCCATGAAGGACCGCCACAGCAAGGTGTGCACGGCGCGCGGGCCGCGGGACCGGCGCGTGCGCCTCTCTGCGCACACCGCCATCCAGTTCTACGACGTGCAGGACCGGCTGGGCTACGACCGCCCCAGCAAGGCCGTCGACTGGCTCATCAAGAACGCCAAGGACGCCATCGACAAGCTCGAGTAGGGCAAGCTTGACCCAGCTTTCTTGTACAAAGTTGGCATTATAAAAAATAATTGCTCATCAATTTGTTGCAACGAACAGGTCACTATCAGTCAAAATAAAATCATTATTTGCCATC |
