# Supplementary material for: Phytoplasma SAP11 effector destabilization of TCP transcription factors differentially impact development and defence of Arabidopsis versus maize
Source: PLoS Pathog. 2019 Sep 26;15(9):e1008035. doi: 10.1371/journal.ppat.1008035 (PMC6802841; doi:10.1371/journal.ppat.1008035)
Supplement: S9 Table — (DOCX) [file ppat.1008035.s022.docx]

**S9 Table.** **Oligonucleotide sequences (5´ > 3´) for qRT-PCR.**

| **Gene** | **Forward primer (5 -> 3)** | **Reverse primer (5 -> 3)** |
| --- | --- | --- |
| *SAP11_AYWB_* | TCTGAAGAGAAGAACAAGAAGCA | AATCATCAGGTTGCTTTGAAGAAG |
| *SAP11_MBSP_* | GAAAGAAGATAGAGGAAAGAATG | GAGTTATCTGGTTTCTTACTAGA |
| *ACTIN2* | GATGAGGCAGGTCCAGGAATC | GTTTGTCACACACAAGTGCATC |
