# Supplementary material for: Phytoplasma SAP11 effector destabilization of TCP transcription factors differentially impact development and defence of Arabidopsis versus maize
Source: PLoS Pathog. 2019 Sep 26;15(9):e1008035. doi: 10.1371/journal.ppat.1008035 (PMC6802841; doi:10.1371/journal.ppat.1008035)
Supplement: S10 Table — Kodzak sequences are in italic, ORFs are flanked by BamHI and EcoR1 restriction sites (grey) for subsequent cloning. (DOCX) [file ppat.1008035.s023.docx]

**S10 Table. Nucleotide sequences for gene syntheses of *FLAG-SAP11_MBSP_* and *FLAG-SAP11_AYWB_* for expression in *Zea mays.*** Kodzak sequences are in italic, ORFs are flanked by *Bam*HI and *Eco*R1 restriction sites (grey) for subsequent cloning.

| ***FLAG-SAP11_AYWB_*** |
| --- |
| GGATCC*CCACC*ATGGACTACAAGGACCACGATGGCGATTACAAGGACCACGACATTGACTACAAGGACGACGACGACAAGAGCCCAAAGAAGGAGAGCTCTGACAAGAAGCGCGATATCCCGAAGATTAACAAGTCAGAGGAGAAGAATAAGAAGCAGAAGGAGGACATCAAGAGGTTCTACACCATTCACAAGGAGTTCAAGGAGTACTCCATCGAGAAGAACAATGAGATCATTAAGATTCTCGAGAACCCGGAGCTCATGGAGATCCTGAAGCAGAAGGCCGAGGAGGAGACGAAGAATCTGAAGGAGGAGGGCTCCAGCTCGAAGCAGCCCGACGATTCGAAGAAGTGAGAATTC |
| ***FLAG-SAP11_MBSP_*** |
| GGATCC*CCACC*ATGGACTACAAGGACCACGATGGCGACTACAAGGACCACGACATTGATTACAAGGACGATGACGACAAGAGCCCCAAGAAGGAGGACCGCGGCAAGAACGTGGCCACCTCCAAGGAGAAGGAGACCCTCACGAAGGAGGAGGTCAAGAGGTTCTTCGAGTACCACAAGACATTCGAGACTTACAGCGACGAGGATAAGATCAAGATCATTGAGAAGATTACGGACCCGGAGGTTTCGAAGCTCCTGGATGAGTACAACGAGAAGAAGCGGAAGTCCAGCAAGGAGGAGTCGTCTTCATCCAAGAAGCCCGACAATTCCAAGAAGTGAGAATTC |
